# Development and validation of a multi-modal ultrasomics model to predict response to neoadjuvant chemoradiotherapy in locally advanced rectal cancer

**DOI:** 10.1186/s12880-024-01237-0

**Published:** 2024-03-18

**Authors:** Qiong Qin, Xiangyu Gan, Peng Lin, Jingshu Pang, Ruizhi Gao, Rong Wen, Dun Liu, Quanquan Tang, Changwen Liu, Yun He, Hong Yang, Yuquan Wu

**Affiliations:** Department of Medical Ultrasound, The First Affiliated Hospital of Guangxi Medical University, No.6 Shuangyong Road, Nanning, Guangxi Zhuang Autonomous Region 530021 China

**Keywords:** Ultrasomics model, Clinical model, LARC, nCRT, Good response

## Abstract

**Objectives:**

To assess the performance of multi-modal ultrasomics model to predict efficacy to neoadjuvant chemoradiotherapy (nCRT) in patients with locally advanced rectal cancer (LARC) and compare with the clinical model.

**Materials and methods:**

This study retrospectively included 106 patients with LARC who underwent total mesorectal excision after nCRT between April 2018 and April 2023 at our hospital, randomly divided into a training set of 74 and a validation set of 32 in a 7: 3 ratios. Ultrasomics features were extracted from the tumors’ region of interest of B-mode ultrasound (BUS) and contrast-enhanced ultrasound (CEUS) images based on PyRadiomics. Mann-Whitney U test, spearman, and least absolute shrinkage and selection operator algorithms were utilized to reduce features dimension. Five models were built with ultrasomics and clinical analysis using multilayer perceptron neural network classifier based on python. Including BUS, CEUS, Combined_1, Combined_2 and Clinical models. The diagnostic performance of models was assessed with the area under the curve (AUC) of the receiver operating characteristic. The DeLong testing algorithm was utilized to compare the models’ overall performance.

**Results:**

The AUC (95% confidence interval [CI]) of the five models in the validation cohort were as follows: BUS 0.675 (95%CI: 0.481–0.868), CEUS 0.821 (95%CI: 0.660–0.983), Combined_1 0.829 (95%CI: 0.673–0.985), Combined_2 0.893 (95%CI: 0.780-1.000), and Clinical 0.690 (95%CI: 0.509–0.872). The Combined_2 model was the best in the overall prediction performance, showed significantly better compared to the Clinical model after DeLong testing (*P* < 0.01). Both univariate and multivariate logistic regression analyses showed that age (*P* < 0.01) and clinical stage (*P* < 0.01) could be an independent predictor of efficacy after nCRT in patients with LARC.

**Conclusion:**

The ultrasomics model had better diagnostic performance to predict efficacy to nCRT in patients with LARC than the Clinical model.

## Introduction

Rectal cancer, one of the most common malignant tumors globally, has the second highest mortality rate among malignant tumors according to the global epidemiological survey in 2020 [1]. Therefore, prompt diagnosis and timely intervention are crucial in mitigating the incidence of relapse and fatality rates associated with rectal cancer. Undergo the total mesorectal excision (TME) after neoadjuvant chemoradiotherapy (nCRT) is the standard treatment for patients with locally advanced rectal cancer (LARC), which induce tumors downsizing and downstaging [2]. However, there are individual differences in tumor response after nCRT, with some patients responding well, and approximately 15–27% of patients can attain a pathological complete response (pCR) after nCRT [3]. About 30–40% of patients have poor response after nCRT, and even a few patients experience tumor progression [4]. Early identification of patients with good response to nCRT can guide treatment strategies to improve quality of life and prognosis. In contrast, patients with poor response to nCRT not only fail to achieve effective control the tumor, but also suffer from nCRT toxicity damage, which can lead to worse quality of life and prognosis [5]. Therefore, it is crucial to explore different predictive methods for early identification of LARC patients with good response after nCRT, which will guide personalized treatment and clinical surgical decision-making for patients.

Recently, multiple studies had reported the predictive performance of CT and MRI radiomics for the efficacy after nCRT in patients with LARC [6–8], while fewer reports of ultrasomics for the efficacy of nCRT in patients with LARC. However, comparing to CT and MRI examinations, ultrasound examination has advantages such as no radiation and short duration. If more diagnostic information can be mined by ultrasomics from ultrasound images to identify LARC patients with good response after nCRT, thus guide patient treatment, this may provide additional value for future multimodal radio studies in rectal cancer. Existing evidence suggested that ultrasomics had a good performance in predicting lymphovascular invasion in rectal cancer [9] and the response of nCRT in breast cancer [10–12]. It had also shown a good diagnostic performance in tumor classification, staging, and differentiation between benign and malignant tumors [13, 14].

The objective of this research is to evaluate the performance of multi-modal ultrasomics models to predict the efficacy in patients with LARC after nCRT and compare with the Clinical model. Univariate and multivariate logistic regression analyses are used to select independent predictors of good response after nCRT in patients with LARC.

## Materials and methods

This retrospective study was conducted with approval from the Medical Ethics Committee of First Affiliated Hospital of Guangxi Medical University (No.2023-E276-01). The requirement of informed consent was waived by the Ethics Committee of First Affiliated Hospital of Guangxi Medical University owing to the retrospective nature of the study. This study was performed in accordance with the ethical standards of the institutional and national research committees as well as the Helsinki Declaration.

### Population

**Fig. 1:**
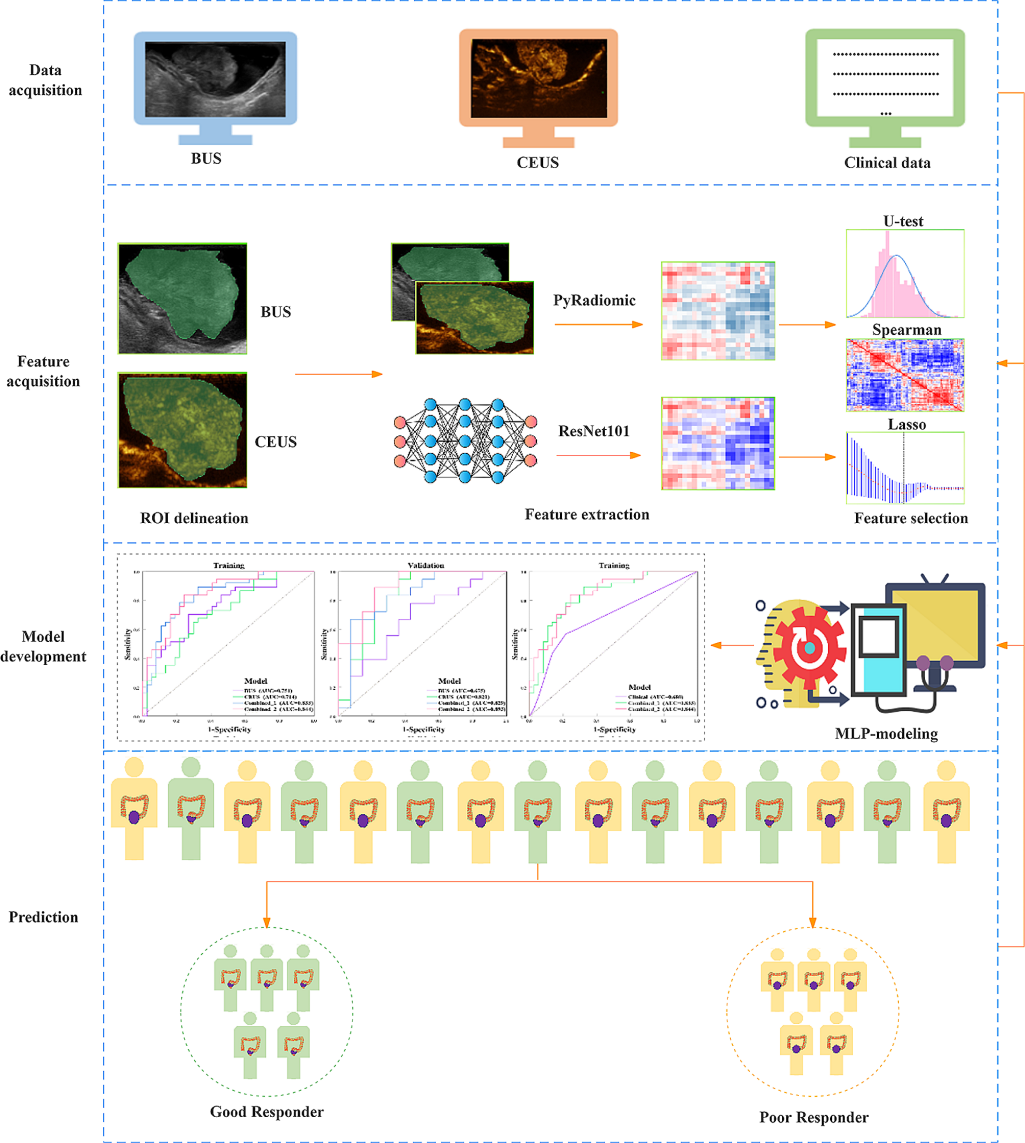
Flowchart of this study Ultrasomics features extracted from the region of interest of tumors based on BUS and CEUS images, respectively. Mann-Whitney U test, spearman, and LASSO were utilized to reduce feature’s dimension. Five models were built based on ultrasomics and clinical analysis using MLP classifier BUS: B-mode ultrasound; CEUS: contrast-enhanced ultrasound; LASSO: least absolute shrinkage and selection operator; MLP: multilayer perceptron neural network

We retrospectively included patients with LARC who underwent TME after nCRT in our hospital from April 2018 to April 2023. Inclusion criteria: (a) patients with LARC received rectal ultrasound examination and had complete B-mode ultrasound (BUS) and contrast-enhanced ultrasound (CEUS) images before nCRT. (b) patients with LARC had completed standard nCRT. (c) complete postoperative pathological data after TME. (d) no prior radiotherapy, chemotherapy or immunotherapy in the past. Exclusion criteria: (a) without TME after nCRT; (b) unable to cooperate to complete ultrasound examination; (c) only with BUS examination without CEUS examination before nCRT; (d) patients with incomplete clinical information. This study design illustrates in Fig. 1.

### Histopathological reference standard

Based on the tumor regression grade (TRG) standard of the eighth edition guide of the AJCC [15]. Two experienced pathologists independently double-blindly classified all patients into four levels and resolved issues by discussion when there was disagreement. TRG was as follows: TRG_0_ indicates a pathological complete response (indicating no viable tumor cells are present). TRG_1_ denotes a near pathological complete response (indicating the presence of single or small groups of tumor cells). TRG_2_ indicates a minimal response (representing the residual cancer outgrown by fibrosis). TRG_3_ indicates an inferior response (representing minimal or no tumor cells were killed).

This study clustered patients into two cohorts: (1) patients with TRG_0 − 1_ were defined as good response; and (2) patients with TRG_2 − 3_ were defined as poor response [16].

### Clinical baseline data

Clinical baseline data of patients before nCRT included age, gender, tumor location, tumor size, clinical stage, T stage, N stage, distant metastasis, CEA, TRG, CA199 and CA242.

### Ultrasound examination protocol

Rectal ultrasound examination was performed using ultrasound diagnostic machines with LOGIQ E9 (IC-5-9-D probe, frequency 5–9 MHz) from General Electric Company in the USA and Mylab Class C (TRT33 probe, convex array mode frequency 3–9 MHz, linear array mode frequency 4–13 MHz) from Italian Esaote. The maximum cross-section of the lesion was first found in grey scale mode and then switched to contrast mode. The ultrasound contrast agent was SonoVue, which was injected with 5 ml of physiological saline before use and configured as a suspension. 2.4 ml SonoVue was quickly injected through the median cubital vein, followed by 5 ml of saline to flush the tube. Synchronous timing and recording, and continuous observation for 3 min. The images were stored and preserved in DICOM format. After offline, the maximum cross-section of the lesion in the early phase of contrast (within 30 s after injection of contrast agent) with the best image quality was selected for subsequent analysis.

### Ultrasomics analysis

A radiologist (Q.Q.) with eight years of experience in rectal ultrasound used ITK-SNAP (version4.0.1; http://www.itksnap.org) to manually segment the region of interest (ROI) of tumors from the BUS and CEUS images, respectively. A senior radiologist confirmed all segmentation masks and any conflicting opinions resolved by discussion. The maximum ROI cross section was extracted from the BUS images and saved in PNG format for further feature extraction with the ResNet101 which was a deep learning model. All the pictures were resampled to a voxel dimension of 1 × 1 × 1 mm. The voxel intensity values were quantized using a bin size of 25 HU [17].

The ultrasomics features were extracted from the BUS and CEUS pictures utilizing PyRadiomics (version 3.0) [18], independently. Which was an open-source software. All features were derived from the initial image and processed images. 1239 features were obtained in all, including 7 types: (1) 17 features of Shape; (2) 247 features of First Order; (3) 182 features for Gray Level Dependence Matrix; (4) 208 features of Gray Level Size Zone Matrix; (5) 65 features of Neighborhood Gray Tone Difference Matrix; (6) 208 features of Gray Level Run Length Matrix; (7) 312 features of Gray Level Co-occurrence Matrix [19]. Additionally, we utilized the pre-trained ResNet101 that a deep learning model to extract 2038 features from the maximum ROI cross-sectional images. After features compression processing, 32 deep learning features were eventually included. All features were standardized using Z-score algorithm to transform them into a uniform measure for comparison.

To avoid overfitting, we employed the Mann-Whitney U-test to choose ultrasomics features with significant distinctions between good response and poor response cohorts. Then spearman’s correlation coefficients were calculated and reserved the features that correlation coefficient > 0.9. To minimize redundancy among radiomic features, we utilized the LASSO (least absolute shrinkage and selection operator) algorithm to reduce features dimension and ten-fold cross validation to select the most relevant features of good response. Built four ultrasomics models using a multilayer perceptron neural network (MLP) classifier based on python, which was an artificial neural network consisting of numerous interconnected neural nodes or layers, where each layer was fully connected to the next layer. Each layer had different weights and was trained using backpropagation. The input layer was responsible for receiving input data, while the output layer produced the final classifications output, and the hidden layers performed different non-linear transformations on the input data. We also used ten-fold cross validation to train the model and selected the optimal parameters to improve model stability. Models were as follows: the BUS model was formed based on only BUS features; the CEUS model was formed based on only CEUS features; the Combined_1 model was created based on the integration of BUS and CEUS features; and the Combined_2 model was created based on the integration of BUS, CEUS, and deep learning features. Additionally, we created a clinical model based on clinical factors.

### Statistical analyses

SPSS software (version 23.0) was employed to process clinical parameters, univariate and multivariate logistic regression analyses were used to select parameters with statistically significant differences between groups. Features selection and models construction performed with Python software (version 3.7). Count data was exhibited with percentage and differences between groups were tested using chi-square test. Metrological data obeying normal distribution was expressed as mean ± standard deviation, and t-test was used for intergroup comparisons; Mann-Whitney U test was used for intergroup comparisons of metrological data obeying skewed distribution. Two-sided *P* values less than 0.05 were considered statistically significant differences. The parameters of diagnostic performance, comprised the area under the curve (AUC 95% confidence interval [CI]), positive predictive value (PPV), and negative predictive value (NPV), specificity, sensitivity, accuracy. The DeLong testing algorithm was utilized to evaluate the overall prediction performance of various models [20].

## Results

### Patient characteristics

**Fig. 2:**
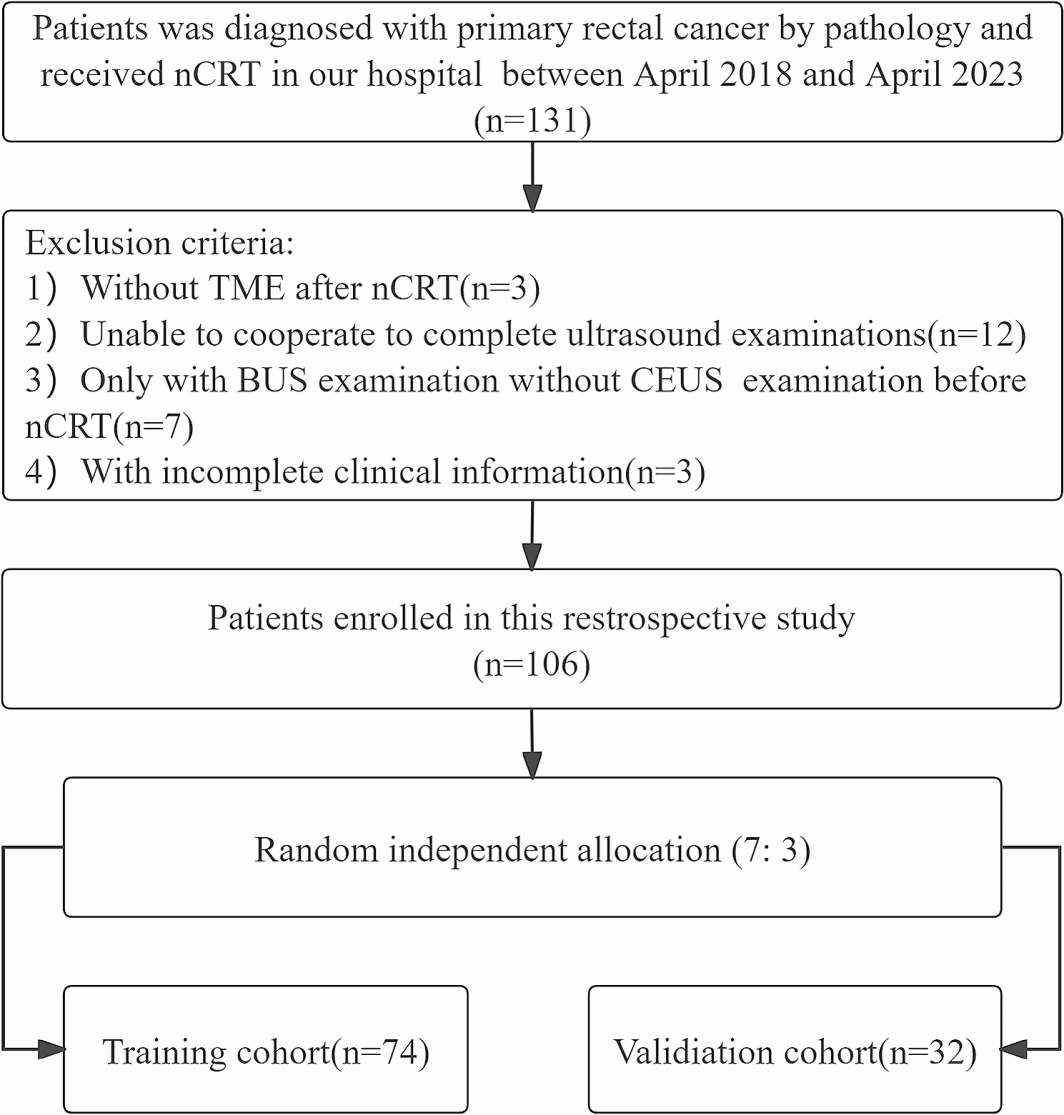
Inclusion and exclusion criteria of patients nCRT: neoadjuvant chemoradiotherapy; TME: total mesorectal excision; BUS: B-mode ultrasound; CEUS: contrast-enhanced ultrasound

This study included 106 patients ultimately, including 51 patients with good response (48.1%) and 55 patients with poor response (51.9%). Patients were randomly divided into a training set 74 and a validation set 32 at 7:3 ratios (Fig. 2), including 72 male (68%) and 34 female (32%) patients, aged 19–78 (mean 54.3 ± 10.0) years.

All clinical pathological parameters, only CEA showed a statistically significant difference between the training set and validation set (*P* < 0.01) (Table 1). Both univariate and multivariate logistic regression analyses showed that age (*P* < 0.01) and clinical stage (*P* < 0.01) could be an independent predictor of good response after nCRT in patients with LARC (Table 2).

**Table 1:** Clinicopathological parameters between training and validation cohorts

Parameters		Training cohort (*n* = 74)	Validation cohort (*n* = 32)	*P*-value
**Sex**				0.739
Male	51(48.11%)		21(19.81%)	
Female	23(21.70%)		11(10.38%)	
**Age**				0.907
≤ 60	54(50.94%)		23 (21.70%)	
> 60	20(18.87%)		9(8.49%)	
**Location (cm)**				0.246
≤ 5	42(39.62%)		22(20.75%)	
5–10	32(30.19%)		10(9.43%)	
**Size (cm)**				0.077
≤ 5	53(50.00%)		28(26.42%)	
> 5	21(19.81%)		4(3.77%)	
**TRG**				0.087
0	19(17.92%)		7(6.60%)	
1	15(14.15%)		10(9.43%)	
2	34(32.08%)		10(9.43%)	
3	6(5.66%)		5(4.72%)	
**Clinical stage**				0.344
II	3(2.83%)		3(2.83%)	
III	55(51.89%)		25(23.58%)	
IV	16(15.09%)		4(3.77%)	
**T stage**				0.881
T__2_	5(4.72%)		3(2.83%)	
T__3_	49(46.23%)		20(18.87%)	
T__4_	20(18.87%)		9(8.49%)	
**N stage**				0.867
N__0_	15(14.15%)		8(7.55%)	
N__1_	19(17.92%)		8(7.55%)	
N__2_	39(36.79%)		15(14.15%)	
N__3_	1(0.94%)		1(0.94%)	
**Distant-metastasis**				0.270
No	58(54.72%)		28(26.42%)	
Yes	16(15.09%)		4(3.77%)	
**CEA (ng/ml)**				0.008
≤ 5	28(26.42%)		21(19.81%)	
> 5	46(43.40%)		11(10.38%)	
**CA199(ng/ml)**				0.529
≤ 37	56(52.83%)		26(24.53%)	
> 37	18(16.98%)		6(5.66%)	
**CA242(U/ml)**				0.613
≤ 20	50(47.17%)		20(18.87%)	
> 20	24(22.64%)		12(11.32%)	

TRG: tumor regression grade; Data in brackets are the percentage, and data outside brackets are the number of patients; Statistical significance was demonstrated by *P* < 0.05

**Table 2:** Univariable and multivariable logistic analysis of the clinical parameters

Parameters	Univariable analysis OR (95%CI)	*P*-value	Multivariable analysis OR (95%CI)	*P*-value
**Age**				
≤ 60 (*n* = 77)	1 [Reference]	NA	1 [Reference]	NA
> 60 (*n* = 29)	3.320 (1.309–8.417)	0.011	3.784 (1.429–10.018)	0.007
**Sex**				
Male(*n* = 72)	1 [Reference]	NA	-	-
Female(*n* = 34)	1.064 (0.470–2.408)	0.881	-	-
**Location (cm)**				
≤ 5(*n* = 64)	1 [Reference]	NA	-	-
5–10(*n* = 42)	1.211(0.555–2.642)	0.631	-	-
**Size (cm)**				
≤ 5(*n* = 81)	1 [Reference]	NA	-	-
> 5(*n* = 25)	1.537(0.618–3.824)	0.355	-	-
**Clinical stage**				
II (*n* = 6)	1 [Reference]	NA	1 [Reference]	NA
III (*n* = 80)	4.000(0,575-27.819)	0.161	6.130 (0.783–47.995)	0.084
IV (*n* = 20)	4.889(1.501–15.924)	0.008	5.459 (1.626–18.328)	0.006
**T stage**				
T_1 − 2_(*n* = 8)	1 [Reference]	NA	-	-
T_3 − 4_(*n* = 98)	1.884(0.427–8.321)	0.403	-	-
**N stage**				
N_0_(*n* = 23)	1 [Reference]	NA	-	-
N_+_(*n* = 83)	1.231(0.488–3.103)	0.660	-	-
**Distant-metastasis**				
No(*n* = 86)	1 [Reference]	NA	-	-
Yes(*n* = 20)	4.821(1.489–15.610)	0.009	-	-
**CEA (ng/ml)**				
≤ 5(*n* = 49)	1 [Reference]	NA	-	-
> 5(*n* = 57)	1.242(0.578–2.669)	0.579	-	-
**CA199 (ng/ml)**				
≤ 35(*n* = 81)	1 [Reference]	NA	-	-
> 35(*n* = 25)	1.006(0.410–2.468)	0.990	-	-
**CA242 (U/ml)**				
≤ 20(*n* = 70)	1 [Reference]	NA	-	-
> 20(*n* = 36)	1.025(0.394–2.667)	0.960	-	-

OR: odds ratio; 95%CI: 95% confidence intervals; NA: not applicable. Data in brackets are the 95% confidence intervals, and data outside brackets are the odds ratio. Statistical significance was demonstrated by *P* < 0.05

### Ultrasomics analysis

After z-score normalization, the BUS model had 1237 features; the CEUS model had 1237 features; the Combined_1 model had 2474 features; and the Combined_2 model had 2506 features. After Mann-Whitney U test, the BUS model had 10 features; the CEUS model had 44 features; the Combined_1 model had 50 features; and the Combined_2 model had 52 features. After spearman correlation analysis, the BUS model had 6 features; the CEUS model had 16 features; the Combined_1 model had 20 features; and the Combined_2 model had 22 features. The final step utilized the LASSO method for features selection, and ultimately, BUS, CEUS, Combined_1, and Combined_2 models incorporated 4, 7, 11, and 12 features respectively to build the ultrasomics models. Table 3 provides information on the included features and corresponds coefficients in every model.

**Table 3:** Features in every model

Model	Selected features	Coefficients
**BUS**	wavelet-LHH_glszm_LargeAreaHighGrayLevelEmphasis_BUS wavelet-LHH_glszm_LargeAreaLowGrayLevelEmphasis_BUS wavelet-HHL_firstorder_Mean_BUS wavelet-HLL_glszm_SmallAreaLowGrayLevelEmphasis_BUS	-0.007677 -0.091875 -0.115225 -0.118419
**CEUS**	wavelet-HLL_glszm_GrayLevelNonUniformityNormalized_CEUS squareroot_glrlm_LongRunEmphasis_CEUS wavelet-HHH_firstorder_Median_CEUS wavelet-HLH_gldm_LowGrayLevelEmphasis_CEUS wavelet-HLH_firstorder_Median_CEUS original_glrlm_ShortRunLowGrayLevelEmphasis_CEUS wavelet-HHH_glszm_SmallAreaHighGrayLevelEmphasis_CEUS	0.06317 0.055382 0.045882 0.012169 -0.03406 -0.076672 -0.155453
**Combined_1**	wavelet-HLL_glszm_GrayLevelNonUniformityNormalized_CEUS wavelet-HLH_gldm_LowGrayLevelEmphasis_CEUS squareroot_glrlm_LongRunEmphasis_CEUS wavelet-HHH_firstorder_Median_CEUS wavelet-HHH_glrlm_RunPercentage_CEUS wavelet-LHH_glszm_LargeAreaLowGrayLevelEmphasis_BUS original_glrlm_ShortRunLowGrayLevelEmphasis_CEUS wavelet-HLH_firstorder_Median_CEUS wavelet-HLL_glszm_SmallAreaLowGrayLevelEmphasis_BUS wavelet-HHL_firstorder_Mean_BUS wavelet-HHH_glszm_SmallAreaHighGrayLevelEmphasis_CEUS	0.076747 0.039037 0.037888 0.029838 0.001871 -0.01021 -0.057341 -0.057341 -0.070581 -0.115704 -0.151225
**Combined_2**	wavelet-HLL_glszm_GrayLevelNonUniformityNormalized_CEUS wavelet-HLH_gldm_LowGrayLevelEmphasis_CEUS squareroot_glrlm_LongRunEmphasis_CEUS wavelet-HHH_firstorder_Median_CEUS original_ngtdm_Coarseness_CEUS wavelet-LHH_glszm_LargeAreaLowGrayLevelEmphasis_BUS original_glrlm_ShortRunLowGrayLevelEmphasis_CEUS wavelet-HLH_firstorder_Median_CEUS resnet101_31 wavelet-HLL_glszm_SmallAreaLowGrayLevelEmphasis_BUS wavelet-HHL_firstorder_Mean_BUS wavelet-HHH_glszm_SmallAreaHighGrayLevelEmphasis_CEUS	0.064434 0.054451 0.039536 0.03321 -0.000446 -0.024238 -0.042904 -0.047193 -0.067187 -0.068875 -0.111608 -0.152471

BUS: B-mode ultrasound; CEUS: contrast-enhanced ultrasound; Combined_1: the integration of BUS and CEUS; Combined_2: the integration of BUS, CEUS, and deep learning

**Fig. 3:**
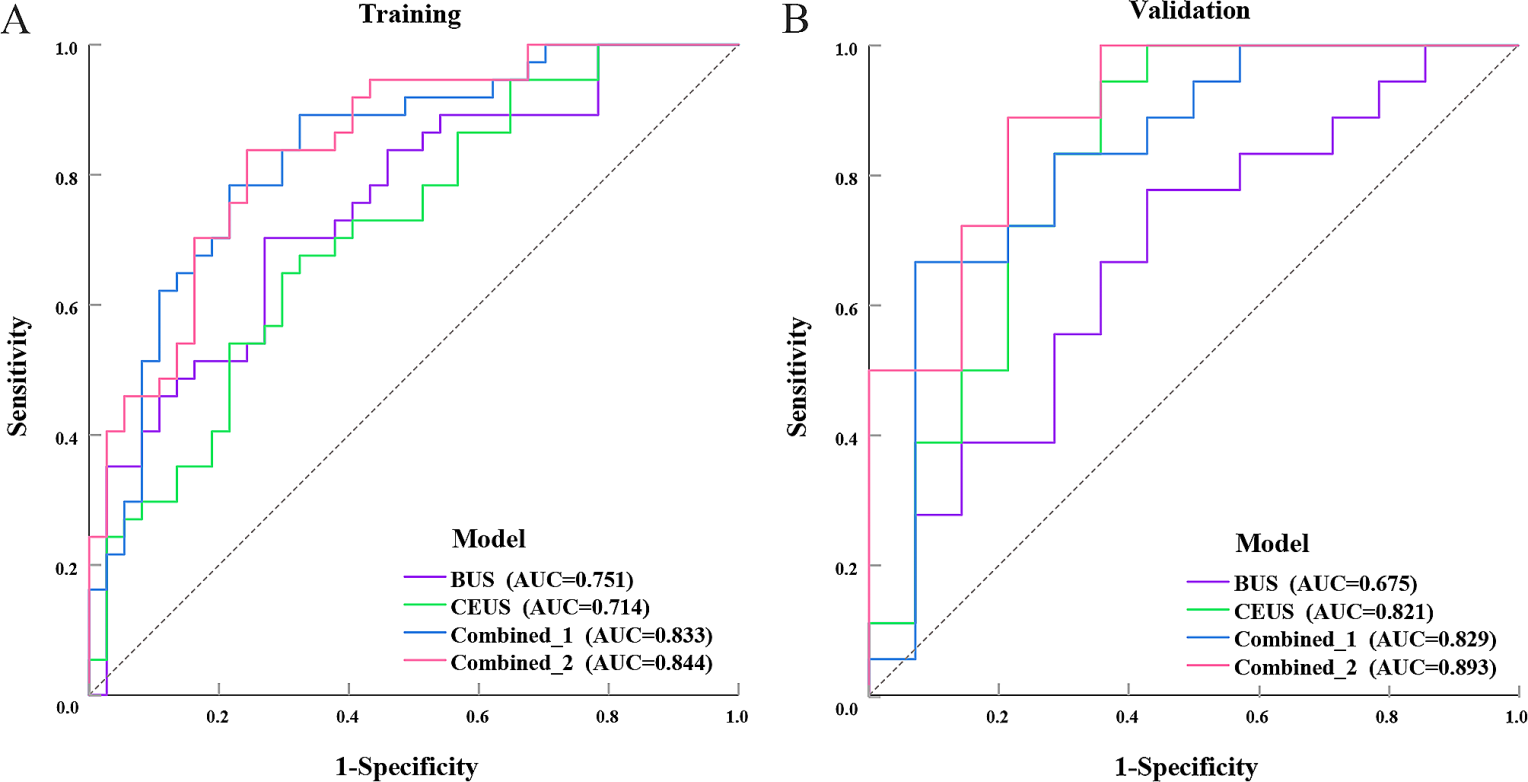
Diagnostic performance of ultrasomics models. (**A**) The ROC curves of four ultrasomics models in training cohort. (**B**) The ROC curves of four ultrasomics models in validation cohort. BUS: B-mode ultrasound; CEUS: contrast-enhanced ultrasound; Combined_1: the integration of BUS and CEUS; Combined_2: the integration of BUS: CEUS: and deep learning; AUC: area under the curve

In the training cohort, sensitivity and specificity were 73.0% and 70.3% for the BUS model, 70.3% and 64.9% for the CEUS model, 67.6% and 89.2% for the Combined_1 model, 75.7% and 83.8% for the Combined_2 model, respectively. In the validation cohort, sensitivity and specificity were 57.1% and 77.8% for the BUS model, 64.3% and 94.4% for the CEUS model, 92.9% and 66.7% for the Combined_1 model, 78.6% and 88.9% for the Combined_2 model, respectively (Table 4). In the training cohort, the AUC of BUS, CEUS, Combined_1, and Combined_2 models to predict good response were 0.751 (95%CI, 0.640–0.862), 0.714 (95%CI, 0.598–0.831), 0.833 (95%CI, 0.740–0.927) and 0.844 (95%CI, 0.757–0.932), respectively. And 0.675 (95%CI, 0.481–0.868), 0.821 (95%CI, 0.660–0.983), 0.829 (95%CI, 0.673–0.985), and 0.893 (95%CI, 0.780-1.000), respectively, in the validation cohort (Table 5; Fig. 3). The outcomes of DeLong testing demonstrated that the prediction performance of the Combined_2 and Combined_1 models regardless of whether it was the training cohort (*P* = 0.774) or the validation cohort (*P* = 0.140) had no significant distinction in statistics (Table 5). Therefore, we would compare these two models with the Clinical model.

**Table 4:** Parameters of diagnostic performance

Model		BUS	CEUS	Combined_1	Combined_2	Clinical
Training cohort	AUC (95%CI)	0.751 (0.640–0.862)	0.714 (0.598–0.831)	0.833 (0.740–0.927)	0.844 (0.757–0.932)	0.680 (0.569–0.792)
	Accuracy (%)	71.6	67.6	78.4	79.7	67.7
	Sensitivity (%)	73.0	70.3	67.6	75.7	78.4
	Specificity (%)	70.3	64.9	89.2	83.8	56.8
	PPV (%)	71.1	66.7	86.2	82.4	64.4
	NPV (%)	72.2	68.6	73.3	77.5	72.4
Validation cohort	AUC (95%CI)	0.675 (0.481–0.868)	0.821 (0.660–0.983)	0.829 (0.673–0.985)	0.893 (0.780-1.000)	0.690 (0.509–0.872)
	Accuracy (%)	68.8	81.2	78.1	84.4	71.9
	Sensitivity (%)	57.1	64.3	92.9	78.6	78.6
	Specificity (%)	77.8	94.4	66.7	88.9	66.7
	PPV (%)	66.7	90.0	68.4	84.6	64.7
	NPV (%)	70.0	77.3	92.3	84.2	80.0

BUS: B-mode ultrasound; CEUS: contrast-enhanced ultrasound; Combined_1: the integration of BUS and CEUS; Combined_2: the integration of BUS, CEUS, and deep learning; AUC: area under the curve; 95% CI: 95% confidence intervals; PPV: positive predictive value; NPV: negative predictive value. Data in brackets are the 95% confidence intervals

**Table 5:** DeLong testing between ultrasomics models

Model		Training cohort					Validation cohort			
		AUC (95%CI)	*P*-value in comparison to BUS	*P*-value in comparison to CEUS	*P*-value in comparison to Combined_1		AUC (95%CI)	*P*-value in comparison to BUS	*P*-value in comparison to CEUS	*P*-value in comparison to Combined_1
BUS		0. 751 (0.640–0.862)	…	0.654	0.078		0.675 (0.481–0.868)	…	0.156	0.025
CEUS		0. 714 (0.598–0.831)	0.654	…	0.024		0.821 (0.660–0.983)	0.156	…	0.905
Combined_1		0.833 (0.740–0.927)	0.078	0.024	…		0.829 (0.673–0.985)	0.025	0.905	…
Combined_2		0.844 (0.757–0.932)	0.141	0.001	0.774		0.893 (0.780-1.000)	0.008	0.107	0.140

BUS: B-mode ultrasound; CEUS: contrast-enhanced ultrasound; Combined_1: the integration of BUS and CEUS; Combined_2: the integration of BUS, CEUS, and deep learning; AUC: area under the curve; 95% CI: 95% confidence intervals. Statistical significance was demonstrated by *P* < 0.05

### Comparison of ultrasomics models and clinical model

**Fig. 4:**
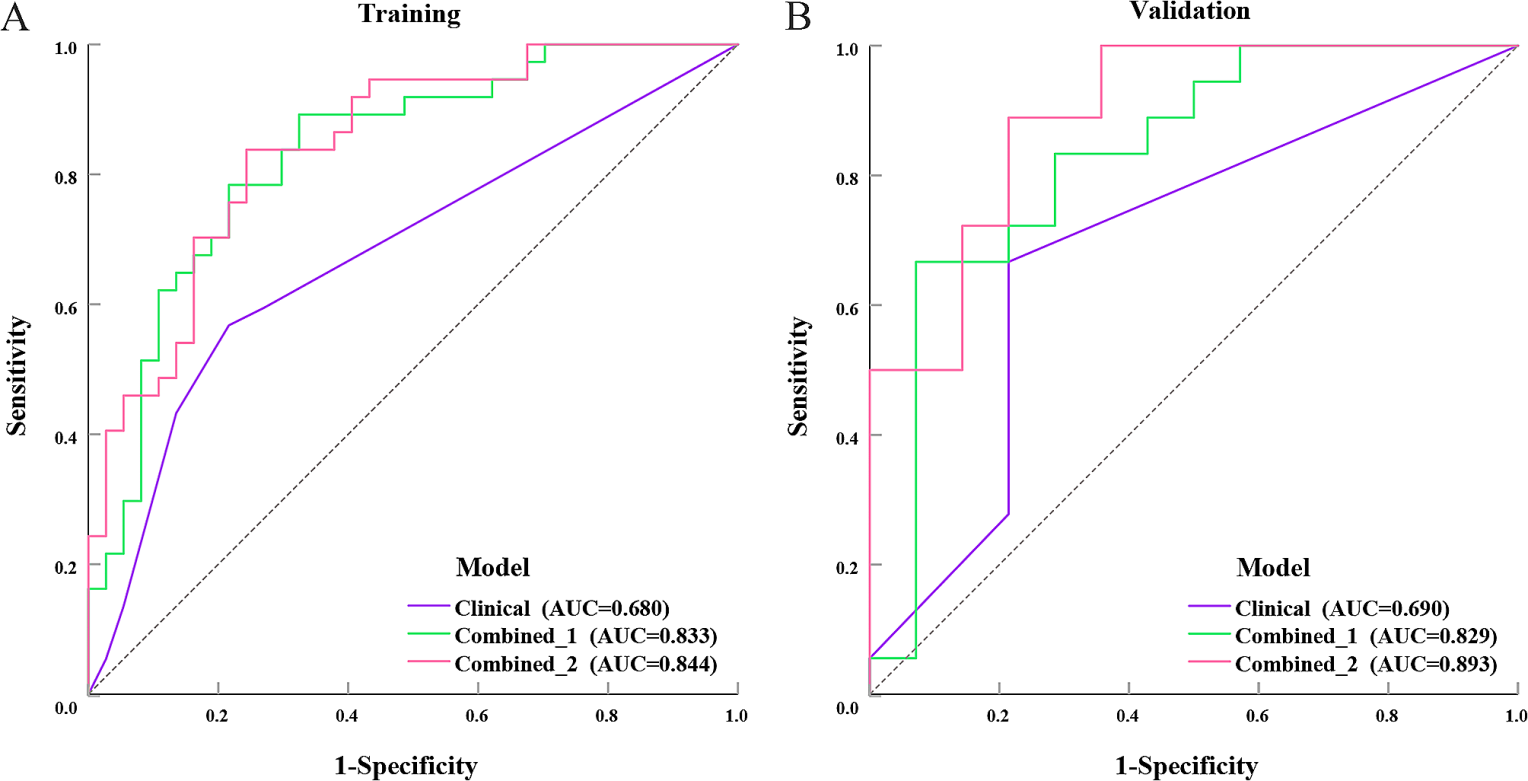
Diagnostic performance of ultrasomics models and clinical model. (**A**) The ROC curves of three models in training cohort. (**B**) **T**he ROC curves of three models in validation cohort. Combined_1: the integration of B-mode ultrasound and contrast-enhanced ultrasound; Combined_2: the integration of B-mode ultrasound: contrast-enhanced ultrasound: and deep learning; AUC: area under the curve

**Fig. 5:**
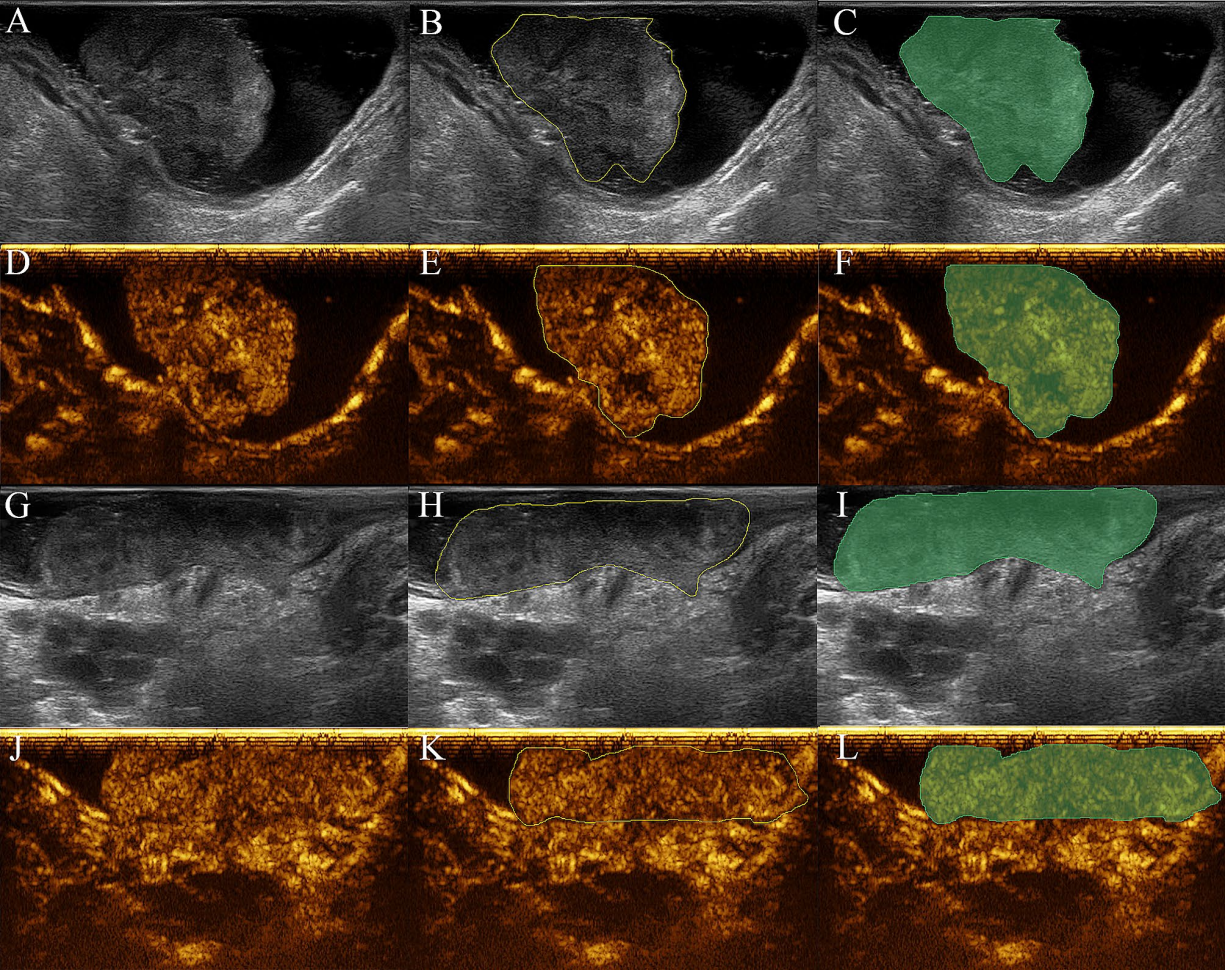
Region of Interest (ROI) for rectal cancer tumors. (**A**-**F**) A patient who had a good response after neoadjuvant chemoradiotherapy. (**G**-**L**) A patient who had a poor response after neoadjuvant chemoradiotherapy. A, G Raw images of tumors from B-mode ultrasound; D, J Raw images of tumors from contrast-enhanced ultrasound. B, E, H, K tracing tumors’ ROI (yellow curve represents the tracing pathway); C, F, I, L Confirming tumors’ ROI (green area)

The AUC for the Combined_1 and Combined_2 models was higher than the Clinical model regardless of whether it was the training group (0.833 [95%CI, 0.740–0.927] for the Combined_1 model and 0.844 [95%CI, 0.757–0.932] for the Combined_2 model vs. 0.680 [95%CI, 0.569–0.792]) or the validation group (0.829 [95%CI, 0.673–0.985] for the Combined_1 model and 0.893 [95%CI, 0.780-1.000] for the Combined_2 model vs. 0.690 [95%CI, 0.509–0.872]) (Table 6; Fig. 4). The results of DeLong testing demonstrated that the AUC of the Combined_2 model was greater than that of the Clinical model regardless of whether it was the training cohort (*P* = 0.007) or the validation cohort (*P* = 0.006) (Table 6). The AUC, accuracy, specificity, and PPV of the Combined_2 model were greater than those of the Clinical model (0.893 vs. 0.690, 84.4% vs. 71.9%, 88.9% vs. 66.7%, 84.6% vs. 64.7%), and the sensitivity and NPV were equivalent between the two models (78.6% vs. 78.6%, 84.2% vs. 80.0%), In the validation cohort (Table 4). Ultrasound images are presented in Fig. 5.

**Table 6:** DeLong testing between ultrasomics models and clinical model

Model		Training cohort				Validation cohort		
		AUC (95%CI)	*P*-value in comparison to Clinical	*P*-value in comparison to Combined_1		AUC (95%CI)	*P*-value in comparison to Clinical	*P*-value in comparison to Combined_1
Clinical		0. 680 (0.561–0.784)	…	0.018		0.690 (0.503–0.841)	…	0.110
Combined_1		0.833 (0.740–0.927)	0.018	…		0.829 (0.673–0.985)	0.110	…
Combined_2		0.844 (0.757–0.932)	0.007	0.774		0.893 (0.780-1.000)	0.006	0.140

Combined_1: the integration of B-mode ultrasound and contrast-enhanced ultrasound; Combined_2: the integration of B-mode ultrasound, contrast-enhanced ultrasound, and deep learning; AUC: area under the curve; 95% CI: 95% confidence intervals. Statistical significance was demonstrated by *P* < 0.05

## Discussion

In this research, we utilized the open-source software PyRadiomics and the pre-trained ResNet101 model to extract features based on BUS and CEUS images. Reduced feature’s dimension gradually and built four ultrasomics models with MLP classifiers. Selected the optimal model through DeLong testing algorithm and compared it with the Clinical model. The Combined_2 model demonstrated the best diagnostic performance among the models in the training group. Produced a better diagnostic performance in assessing good response compared to the Clinical model (AUC, 0.893[95%CI: 0.780-1.000] vs. 0.690[95%CI: 0.509–0.872]), with greater in accuracy (84.4% vs. 71.9%), specificity (88.9% vs. 66.7%) and PPV (84.6% vs. 64.7%), had no significant difference in sensitivity (78.6% vs. 78.6%) and NPV (84.2% vs. 80.0%). The advantage of this study is that using baseline data before nCRT to construct models to predict the efficacy after nCRT, which will guide clinical surgical decision-making and personalized treatment for patients.

In recent years, there had been frequent reports on the prediction of nCRT efficacy in patients with LARC based on radiomics, mostly based on single or multiple MRI sequences [15, 21–25]. It is worldwide accepted that MRI is the gold standard and patients require MRI after completion of CRT not only to assess response but also resectability. It has also been proven effective and robust in assessing residual disease and guiding patients to choose a watch-and-wait treatment strategy, and predicting survival as correlates with histopathology findings. However, there are very few reports on the prediction of nCRT in patients with LARC based on ultrasomics. This may be related to the difficulty in achieving the satisfactory quality of ultrasound images: firstly, the acquisition of each ultrasound image needs to be manually completed by the examining physician; Secondly, optimal imaging parameters vary for each patient; Finally, the patient’s own conditions and cooperation also influence the quality of the images. Ultrasound examination also has certain advantages, such as it is radiation-free and imaging time is significantly shorter than MRI and so on. This study constructs predictive models based on multimodal ultrasound images, and the results show that the transfer learning models based on BUS and CEUS have high diagnostic efficiency for identifying LARC patients with good response to nCRT. This may provide additional value for future multimodal radio studies in rectal cancer. Interestingly, a recent study reported the prediction of radiotherapy response in patients with rectal cancer based on transrectal ultrasound, which found that ultrasomics scores could serve as a biomarker to predict the pathological characteristics of rectal cancer [26]. Unfortunately, this study did not use DeLong test to compare the diagnostic performance between models, and also had a small sample size of only 43 cases. Our study had a relatively larger sample size than this study (106 vs. 43).

Previous studies had indicated that various clinical factors could affect the pathological reaction of nCRT in patients with LARC, including the CEA and CA199 level, and clinical staging [27, 28]. In our investigation, both univariate and multivariate logistic regression analyses show that age (*P* < 0.01) and clinical stage (*P* < 0.01) can be as an independent predictor of good response after nCRT in patients with LARC. It demonstrates that patients over 60 years of age and stage IV clinical staging, have a poorer response to nCRT, which is consistent with the findings of previous research. The AUC value of the clinical model was 0.690 in our study, which was consistent with the outcomes of previous research [29]. However, the diagnostic performance of the clinical model was significantly lower than the ultrasomics model in our study (AUC, 0.690 vs. 0.893, *P* < 0.01).

Comparing the prediction performance of four ultrasomics models through DeLong testing, which proves that the Combined_1 model shows greater than the BUS model in the validation cohort (*P* < 0.05) and the CEUS model in the training set (*P* < 0.05). Interestingly, although the AUC value of the Combined_2 model is superior than the Combined_1 model, its overall performance has no significant advantages regardless of whether it is the training group (AUC, [0.844 vs. 0.833, *P* = 0.774]) or the validation group (AUC, [0.893 vs. 0.829, *P* = 0.140]). This may be related to the pre-trained ResNet101 model. If we use a deep learning model that trained with our own images for transfer learning, its performance may be significantly improved, which is what we wanted to achieve in the future.

Although the results of this study are promising, there are several constraints: firstly, this study is a single-center retrospective study, which may result in selection bias and lack of generalizability. Prospective studies, multi- center data integration and external validation are essential in the future. Secondly, although we have adopted “ten-fold cross validation” to improve the model’s stability, the limited sample size still has an effect on it. Expanding the sample size to train the model is expected to improve its stability and increase clinical applicability in the future. Thirdly, we only use the pre-nCRT data, we will combine post-nCRT data to train the model in the future, which is expected to improve its predictive performance. Fourthly, we only use the pre-trained model to extract deep learning features. It is necessary to use deep transfer learning model to extract features to train the model in the future. Finally, we only use ultrasound images, and in the future, integrating multimodal data such as CT and MRI images to train the model is expected to improve its predictive performance.

## Conclusion

In conclusion, the ultrasomics models show higher diagnostic performance than the clinical model to predict good response in patients with LARC after nCRT. This study indicates that ultrasomics scores have the prospective to become a non-invasive radiomics biomarker to predict efficacy in patients with LARC after nCRT in the future. It will provide valuable information for surgical decision-making by clinicians and personalized treatment for patients.

## Data Availability

All data generated or analysed during this study are included in this published article.

## Abbreviations

AUC: Area under the curve;
BUS: B-mode ultrasound;
CEUS: Contrast-enhanced ultrasound;
CI: Confidence interval;
LARC: Locally advanced rectal cancer;
LASSO: Least absolute shrinkage and selection operator;
MLP: Multilayer perceptron neural network;
nCRT: Neoadjuvant chemoradiotherapy;
NPV: Negative predictive value
pCR: Pathological complete response;
PPV: Positive predictive value;
ROI: Region of interest;
TRG: Tumor regression grade;
